# Predicting feature genes correlated with immune infiltration in patients with abdominal aortic aneurysm based on machine learning algorithms

**DOI:** 10.1038/s41598-024-55941-6

**Published:** 2024-03-02

**Authors:** Yufeng Zhang, Gang Li

**Affiliations:** 1 Department of Vascular Surgery, The Second Affiliated Hospital of Shandong First Medical University, Tai’an, 271000 Shandong China; 2 Postdoctoral Workstation, Shandong First Medical University & Shandong Academy of Medical Sciences, Jinan, 250021 Shandong China; 3 Department of Pulmonary and Critical Care Medicine, Jiangyin Hospital of Traditional Chinese Medicine, Jiangyin Hospital Affiliated to Nanjing University of Chinese Medicine, Jiangyin, 214400 Jiangsu China

**Keywords:** Cardiovascular diseases, Computational biology and bioinformatics, Biomarkers

## Abstract

Abdominal aortic aneurysm (AAA) is a condition characterized by a pathological and progressive dilatation of the infrarenal abdominal aorta. The exploration of AAA feature genes is crucial for enhancing the prognosis of AAA patients. Microarray datasets of AAA were downloaded from the Gene Expression Omnibus database. A total of 43 upregulated differentially expressed genes (DEGs) and 32 downregulated DEGs were obtained. Function, pathway, disease, and gene set enrichment analyses were performed, in which enrichments were related to inflammation and immune response. *AHR*, *APLNR*, *ITGA10* and *NR2F6* were defined as feature genes via machine learning algorithms and a validation cohort, which indicated high diagnostic abilities by the receiver operating characteristic curves. The cell-type identification by estimating relative subsets of RNA transcripts (CIBERSORT) method was used to quantify the proportions of immune infiltration in samples of AAA and normal tissues. We have predicted *AHR*, *APLNR*, *ITGA10* and *NR2F6* as feature genes of AAA. CD8 + T cells and M2 macrophages correlated with these genes may be involved in the development of AAA, which have the potential to be developed as risk predictors and immune interventions.

## Introduction

Abdominal aortic aneurysm (AAA) is a condition characterized by a pathological and progressive dilatation of the infrarenal abdominal aorta, resulting in an elevated rupture risk^1^. Mortality in ruptured AAA can be as high as 85%^2^. AAA has become a serious life-threatening disease due to a lack of insight into its pathogenesis and early intervention^3^. The paucity of effective drug therapies emphasizes the urgent need for a deeper comprehension of the molecular and cellular mechanisms underlying AAA onset and progression^4^. Therefore, the exploration of AAA feature genes is crucial for enhancing the prognosis of patients with AAA.

Several genes and biomarkers have been identified in multiple studies to be crucial in the pathogenesis of AAA^5–7^. AAA is a complex disease caused by a confluence of multiple factors including environmental, biochemical, and genetic variables^8^. The main pathophysiologic mechanisms of AAA include extracellular matrix degradation, depletion of vascular smooth muscle cells, oxidative stress, and inflammatory cell infiltration^9,10^. Inflammatory immune cell infiltration is identified as a driver of AAA development^11,12^.

Recently, microarray technology and machine learning algorithms have been combined to determine genes correlated with various disorders that may be feature genes^13,14^. Additionally, researches have revealed that these genes are highly associated with immunological infiltration, which is becoming more and more significant^15,16^. However, to date, few studies have explored immune infiltration and potential feature genes in AAA by applying microarray methods and machine learning algorithms. Relying on the strong classification and prediction ability of machine learning, the analysis from the big data of microarray can be more accurately to screen out of the feature genes.

The Gene Expression Omnibus (GEO) database was initially accessed in this study to obtain AAA microarray datasets. A metadata cohort was generated by integrating two datasets. Aorta samples from patients suffering from AAA as well as normal control samples were used to identify differentially expressed genes (DEGs). Thereafter, Gene Ontology (GO) functional enrichment analysis, Kyoto Encyclopedia of Genes and Genomes (KEGG) pathway enrichment analysis, Disease Ontology (DO) enrichment analysis, and gene set enrichment analysis (GSEA) were carried out. Then, candidate feature genes of AAA were filtered and identified via machine learning algorithms. An additional validation cohort from the GEO database was used to confirm the validity of these genes. The prediction ability of the identified feature genes in the metadata and validation cohorts was examined via receiver operating characteristic (ROC) curves. The levels of immune cell infiltration in samples of AAA and normal controls were eventually quantified with the cell-type identification by estimating relative subsets of RNA transcripts (CIBERSORT) approach as per their gene expression patterns. We also delved into the association between feature genes and immune cell infiltration, laying the groundwork for further investigation.

## Results

### DEG identification

**Figure 1:**
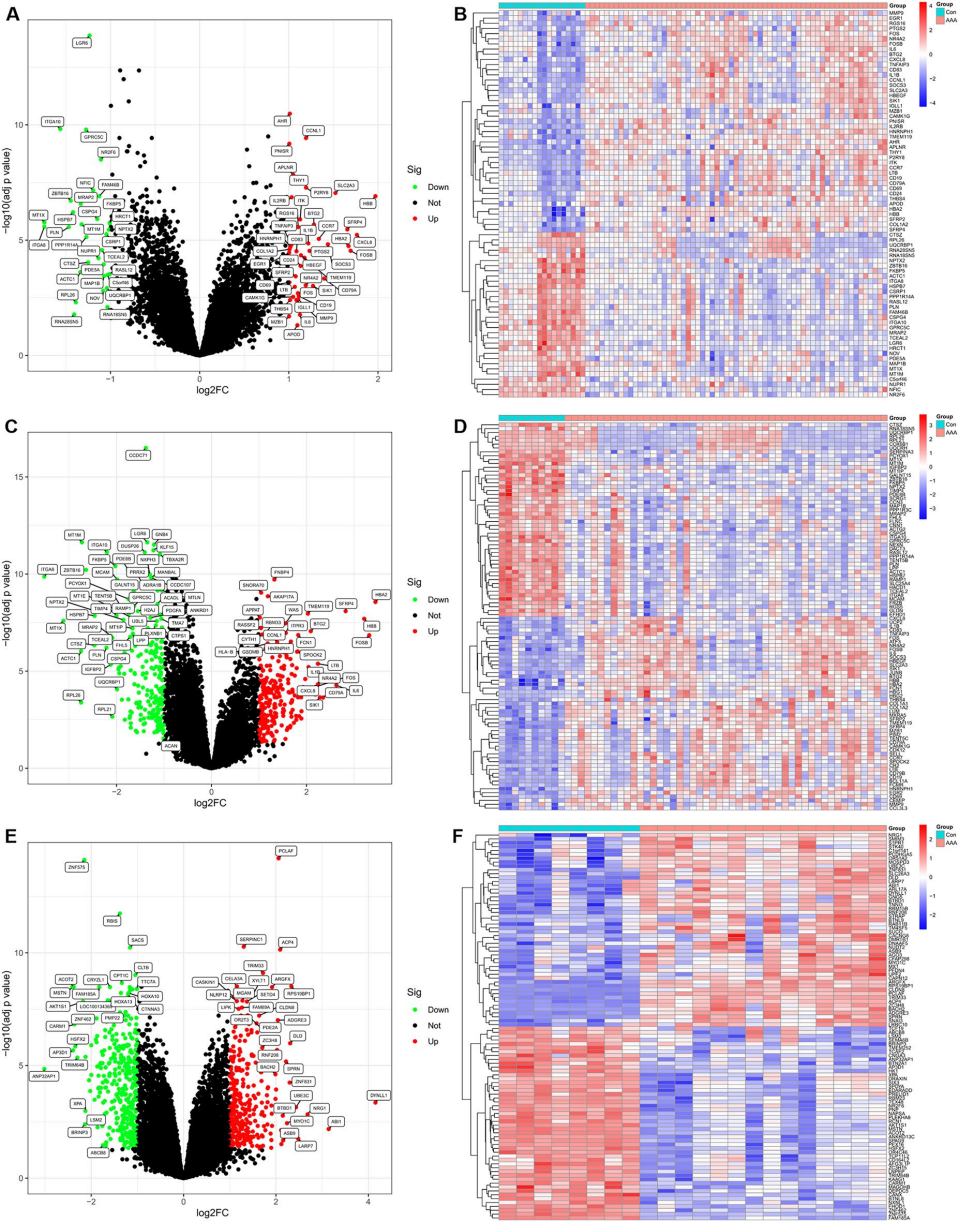
DEGs between AAA and control samples. (**A**) The volcano plot of the DEGs in the metadata cohort. (**B**) The expression profiles of the DEGs in the metadata cohort are displayed on the heatmap. (**C**) The volcano plot of the DEGs in GSE57691. (**D**) The expression profiles of the DEGs in GSE57691 are displayed on the heatmap. (**E**) The volcano plot of the DEGs in GSE47472. (**F**) The expression profiles of the DEGs in GSE47472 are displayed on the heatmap. Volcano plot: the thresholds were established at |log2FC|> 1 and adj p < 0.05; the genes upregulated and downregulated in the AAA samples are shown by the red (Up) and green (Down) dots respectively; genes that do not exhibit a difference in expression between the AAA and normal samples are represented by the black dots (Not). Heatmap: control samples (Con) and AAA samples (AAA) showed varied expression levels. Blue denotes low expression, whereas red denotes high expression.

The non-normalized raw data were downloaded from two GEO datasets (GSE57691 and GSE47472). The clinical overall design and sample information of GSE57691 and GSE47472 patient cohorts is shown in Supplementary File 1A–C. The samples from patients suffering from AAA or control aorta samples from organ donors were the inclusion criteria for the sample selection in the datasets. The GSE57691 dataset contained 10 control aorta samples from organ donors and 49 aorta samples from patients suffering from AAA. The GSE47472 dataset contained aorta samples taken from eight control organ donors and 14 patients with AAA. Then, the expression matrices were obtained using the “lumi” package and gene symbols were created for each probe in each dataset utilizing the probe annotation files (see Supplementary File 2A,B). The two expression matrices were merged into one expression matrix of 63 AAA and 18 control samples using the “SVA” package (see Supplementary File 2C). The expression and principal component analysis (PCA) before and after batch correction showed that the baseline of the two datasets were consistent and further normalized (**see** Supplementary Fig. S1). After eliminating the batch effects, the “limma” package was utilized to analyze the DEGs between AAA and control samples in the metadata cohort. In total, 75 DEGs were identified when adjusted (adj) P value < 0.05 and |log2 fold change (FC)|> 1 were chosen as the cutoff criteria. Of these, 43 were upregulated (log2FC > 1) and 32 were downregulated (log2FC < − 1) genes (see Supplementary File 3A). Figure 1A shows the volcano plot of the DEGs while Fig. 1B shows a heatmap showing the expression levels of DEGs in the metadata cohort. Correspondingly, we also analyzed the DEGs between AAA and control samples in the individual datasets (GSE57691 and GSE47472) (see Supplementary File 3B,C). Compared with a single dataset, by increasing the sample size, we found the DEGs are more accurate in the metadata cohort. Figure 1C shows the volcano plot of the DEGs while Fig. 1D shows a heatmap showing the expression levels of DEGs in GSE47472. Figure 1E shows the volcano plot of the DEGs while Fig. 1F shows a heatmap showing the expression levels of DEGs in GSE57691.

### GO functional and KEGG pathway enrichment analyses

**Figure 2:**
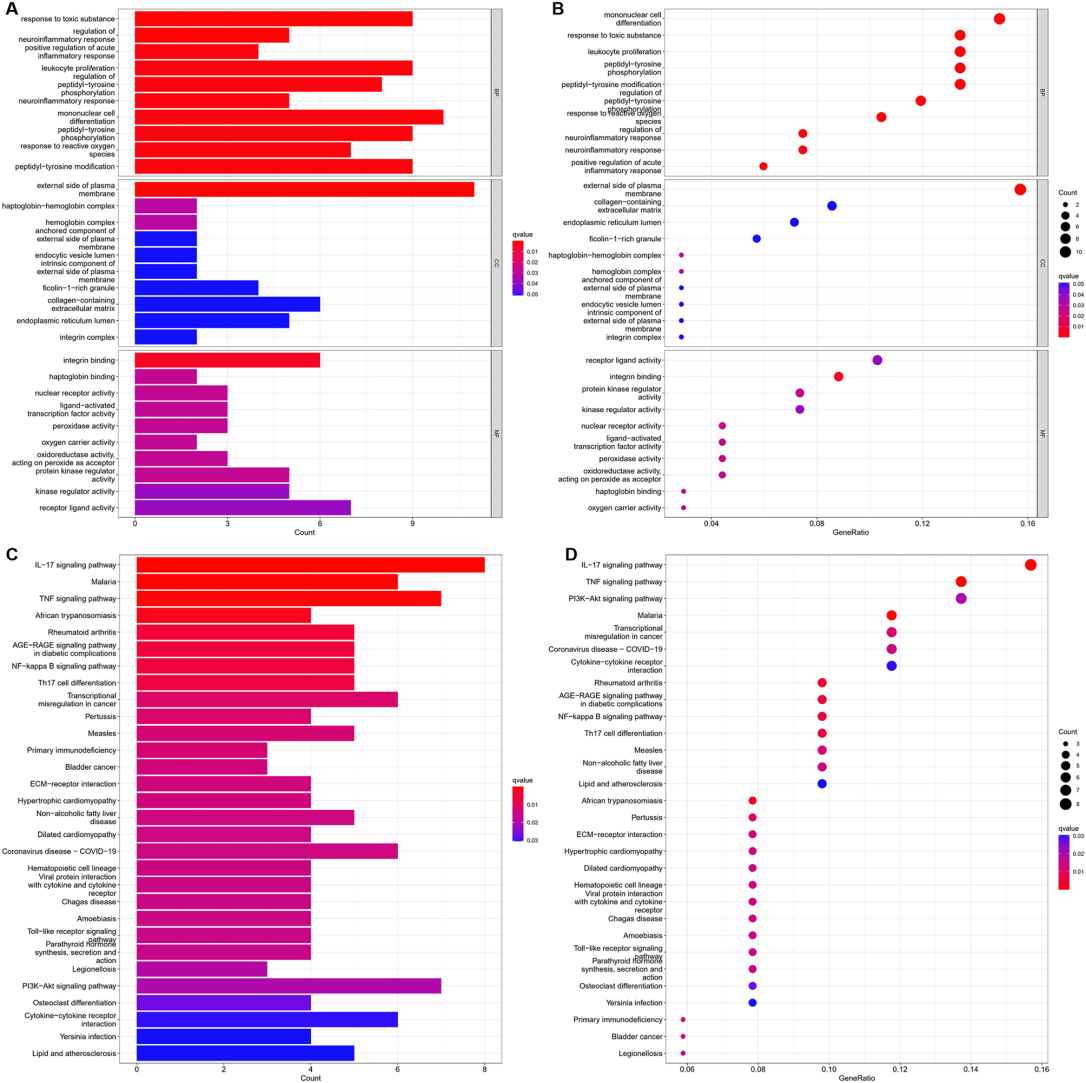
GO functional and KEGG pathway enrichment analyses. (**A**) Ranking of the top 10 GO functional enrichments according to q value. (**B**) Ranking of the top 10 GO functional enrichments according to the count value. (**C**) The top 30 of the most enriched KEGG pathways, ordered by q value. (**D**) The top 30 of the most enriched KEGG pathways, ordered by count value. *BP* biological process, *CC* cellular component, *MF* molecular function.

The findings from GO biological process (BP) enrichment analysis demonstrated significant enrichment of DEGs in response to toxic substances, regulation of neuroinflammatory response, positive regulation of acute inflammatory response, response to reactive oxygen species, leukocyte proliferation, regulation of peptidyl-tyrosine phosphorylation, mononuclear cell differentiation, peptidyl-tyrosine phosphorylation, neuroinflammatory response, peptidyl-tyrosine modification among others. The findings of GO cellular component (CC) enrichment analysis demonstrated significant enrichment of DEGs in external side of plasma membrane, haptoglobin-hemoglobin complex, and hemoglobin complex. The findings of GO molecular function (MF) enrichment analysis demonstrated significant enrichment of DEGs in integrin binding, haptoglobin binding, nuclear receptor activity, ligand-activated transcription factor activity, peroxidase activity, oxygen carrier activity, oxidoreductase activity acting on peroxide as acceptor, protein kinase regulator activity, kinase regulator activity, and receptor ligand activity among others (see Supplementary File 4A). As presented in Fig. 2A,B, the top 10 GO functional enrichments are displayed in order of q value and count value, respectively. The GO plots represented as a network to understand the connection between most prominent GO terms were shown in Supplementary Fig. S2.

The KEGG pathway enrichment demonstrated significant enrichment of DEGs in interleukin 17 (IL-17) signaling pathway, T helper 17 (Th17) cell differentiation, malaria, African trypanosomiasis, nuclear factor κB (NF-κB) signaling pathway, rheumatoid arthritis, advanced glycation end products (AGEs)-receptor for AGE (RAGE) signaling pathway in diabetic complications, tumor necrosis factor (TNF) signaling pathway, transcriptional misregulation in cancer, pertussis among others (see Supplementary File 4B). Figure 2C,D present the top 30 KEGG pathway enrichments ranked by q value and the top 30 KEGG pathway enrichments ranked by count value, respectively.

### DO enrichment analysis and GSEA

**Figure 3:**
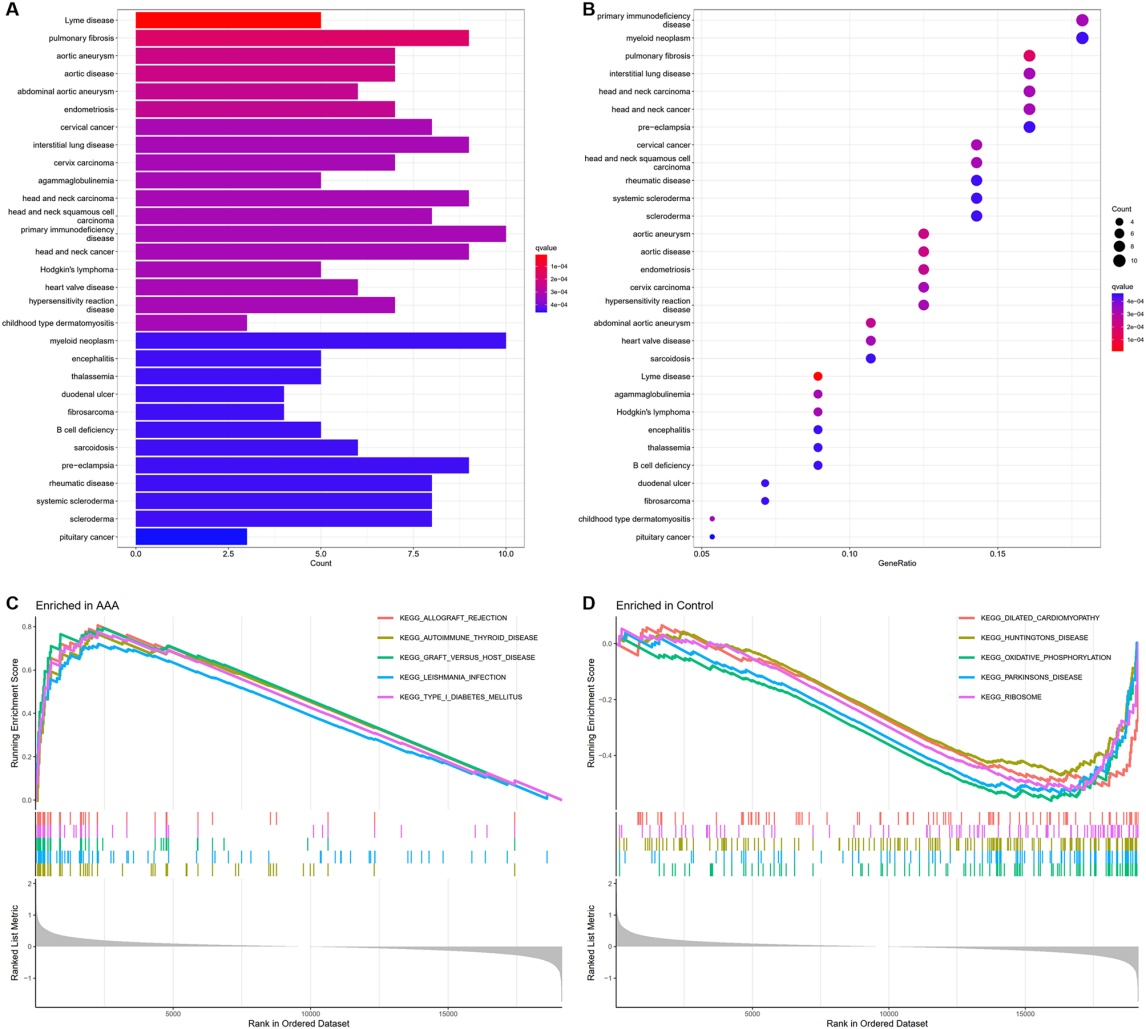
DO enrichment analysis and GSEA of DEGs. (**A**) Ranking of the top 30 DO enrichments according to q value. (**B**) Ranking of the top 30 DO enrichments by the count value. (**C**) Gene set enrichments at the top of the ranked list. The five gene set enrichments at the top of the ranked list (NES > 1) indicate higher expression in AAA. (**D**) Enrichments of gene sets are at the bottom of the sorted list. The five gene set enrichments at the bottom of the ranked list (NES < − 1) indicate lower expression in AAA.

The diseases enriched by DEGs was done using DO enrichment analysis. The findings showed that a range of disorders was primarily linked to those enriched by DEGs, such as Lyme disease, pulmonary fibrosis, aortic aneurysm, aortic disease, AAA, endometriosis, cervical cancer, interstitial lung disease, cervix carcinoma and agammaglobulinemia among others (see Supplementary File 4C). In Fig. 3A,B, the top 30 DO enrichments by q value and the top 10 by count value are displayed, respectively.

According to the GSEA data, the enriched pathways were primarily involved in allograft rejection, Huntington’s disease, dilated cardiomyopathy, type I diabetes mellitus, oxidative phosphorylation, leishmania infection, Parkinson’s disease, autoimmune thyroid disease, graft versus host disease, ribosomes and so on (see Supplementary File 4D). The enrichment score (ES) measures the degree to which a gene set is overrepresented at the top or bottom of a ranked list of genes. According to normalized ES (NES) value, gene set enrichments at the top of the ranked list (NES > 1) indicate higher expression in AAA and gene set enrichments at the bottom of the ranked list (NES < − 1) indicate lower expression in AAA. Figure 3C displays the top five gene set enrichments by p value from the list (NES > 1). The five gene set enrichments at the bottom of the ranked list ranked by p value (NES < − 1) are shown in Fig. 3D.

### Identification of candidate feature genes

**Figure 4:**
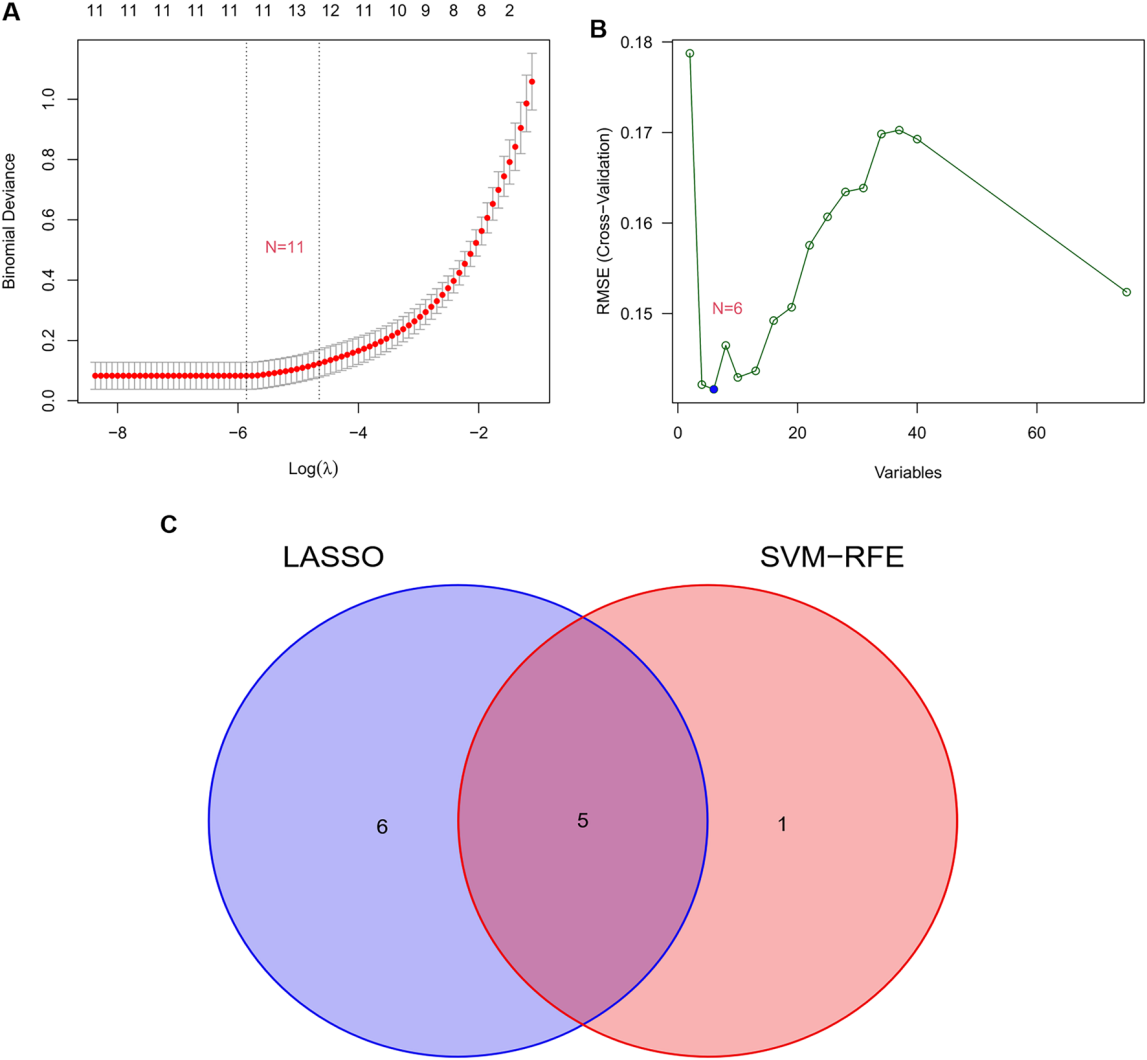
Screening candidate feature genes using two machine learning algorithms. (**A**) Using the LASSO method to select feature genes. (**B**) A plot showing the feature genes chosen by the SVM-RFE method. (**C**) Five feature genes matched by the LASSO and SVM-RFE methods are shown in a Venn diagram. The five overlapping genes (*AHR*, *ITGA10*, *PNISR*, *NR2F6*, and *APLNR*) between the two algorithms were selected.

The candidate feature genes were screened using two algorithms. Eleven variables were found to be candidate feature genes for AAA after the DEGs were reduced utilizing the least absolute shrinkage and selection operator (LASSO) regression model (Table 1, Fig. 4A). The support vector machine (SVM)-recursive feature elimination (RFE) algorithm was employed to discover a subset of six genes from the DEGs (Table 2, Fig. 4B). The five overlapping genes between the two algorithms were ultimately selected as follows, *AHR*, *ITGA10*, *PNISR*, *NR2F6*, and *APLNR*, which were defined as candidate feature genes (Fig. 4C).

**Table 1:** Identification of 11 genes using the LASSO regression algorithm.

Gene symbol	Description
*AHR*	Aryl Hydrocarbon Receptor
*ITGA10*	Integrin Subunit Alpha 10
*PNISR*	PNN Interacting Serine and Arginine Rich Protein
*NR2F6*	Nuclear Receptor Subfamily 2 Group F Member 6
*APLNR*	Apelin Receptor
*THY1*	Thy-1 Cell Surface Antigen
*NFIC*	Nuclear Factor I C
*SLC2A3*	Solute Carrier Family 2 Member 3
*HBB*	Hemoglobin Subunit Beta
*COL1A2*	Collagen Type I Alpha 2 Chain
*RNA28SN5*	RNA, 28S Ribosomal N5

**Table 2:** Identification of six genes using the SVM-RFE algorithm.

Gene symbol	Description
*NR2F6*	Nuclear Receptor Subfamily 2 Group F Member 6
*LGR6*	Leucine Rich Repeat Containing G Protein-Coupled Receptor 6
*AHR*	Aryl Hydrocarbon Receptor
*PNISR*	PNN Interacting Serine And Arginine Rich Protein
*ITGA10*	Integrin Subunit Alpha 10
*APLNR*	Apelin Receptor

### Validation of feature genes

**Figure 5:**
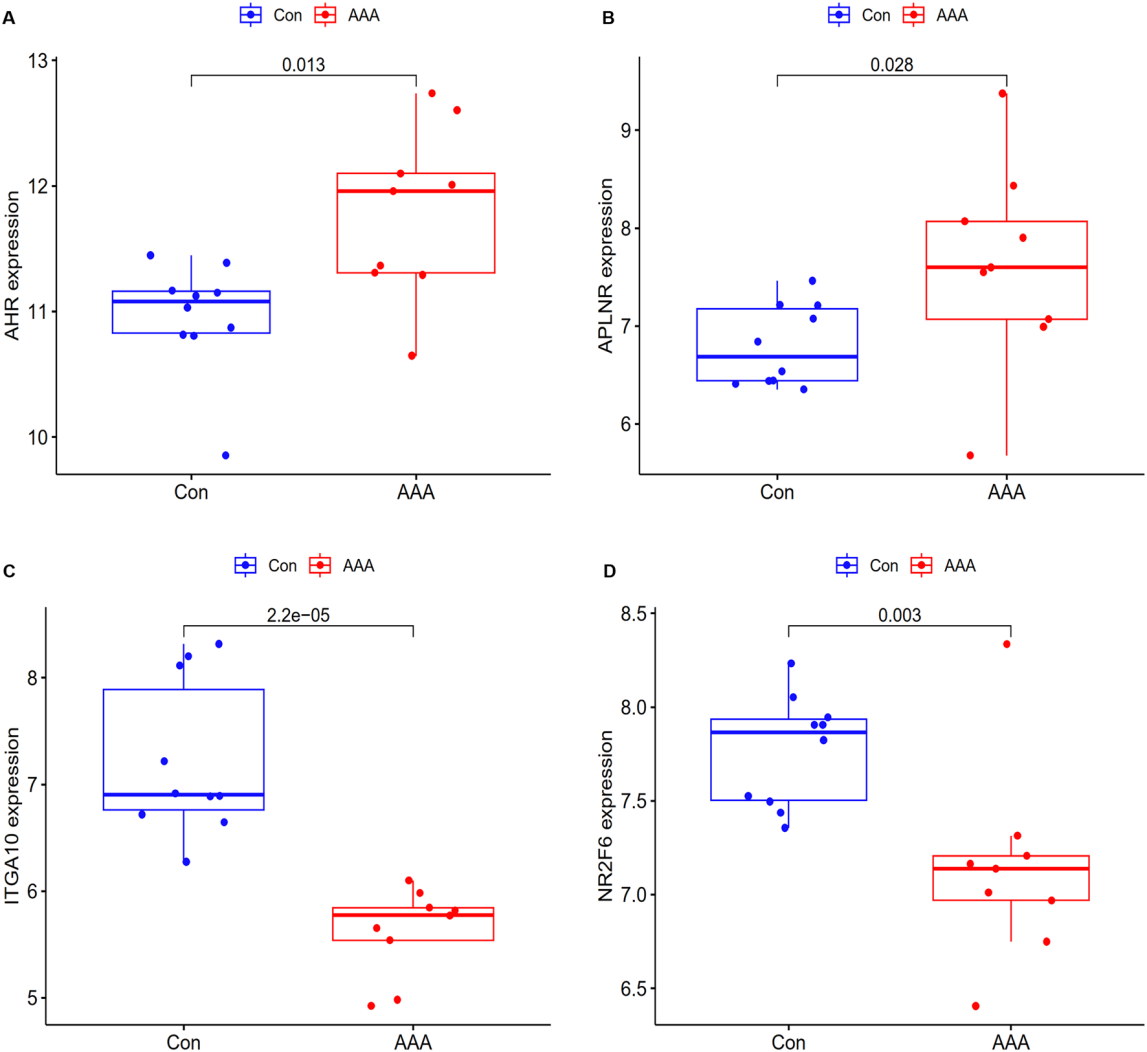
Validation of the expression of feature genes in the GSE7084 dataset. Comparison of the gene expression levels in AAA (AAA) samples to those in control samples (Con). (**A**) *AHR* was expressed at significantly higher levels in AAA samples relative to control samples. (**B**) In comparison to the control samples, the levels of *APLNR* expression in AAA samples were substantially elevated. (**C**) When compared to the control samples, the levels of *ITGA10* expression in AAA samples were considerably lower. (**D**) Comparing AAA samples to the control samples, the expression levels of *NR2F6* were remarkably lower in the former.

Additionally, the GSE7084 dataset was examined to validate the expression patterns of the five genes to provide highly accurate and robust outcomes. The clinical overall design and sample information of GSE7084 patient cohort is shown in Supplementary File 1A,D. Given that the GSE7084 dataset was based on two platforms, the “SVA” package was also used to construct an expression matrix from nine AAA samples and 10 control samples (see Supplementary File 5). In comparison to the control samples, the levels of *AHR* expression in AAA samples were substantially elevated (p = 0.013) (Fig. 5A). *APLNR* was expressed at significantly greater levels in AAA samples compared to control samples (p = 0.028) (Fig. 5B). As opposed to the control samples, the *ITGA10* expression levels in AAA samples were markedly lower (p < 0.001) (Fig. 5C). The expression levels of *NR2F6* in AAA samples were remarkably lower than those in the control samples (p = 0.003) (Fig. 5D). The differential expression of these genes in the metadata cohort was supported by these findings. Nevertheless, *PNISR* expression levels did not differ significantly across AAA and control samples (see Supplementary Fig. S3). Therefore, the four selected genes (*AHR*, *APLNR*, *ITGA10*, and *NR2F6*) were identified as feature genes for further study.

### Diagnostic performance of feature genes

**Figure 6:**
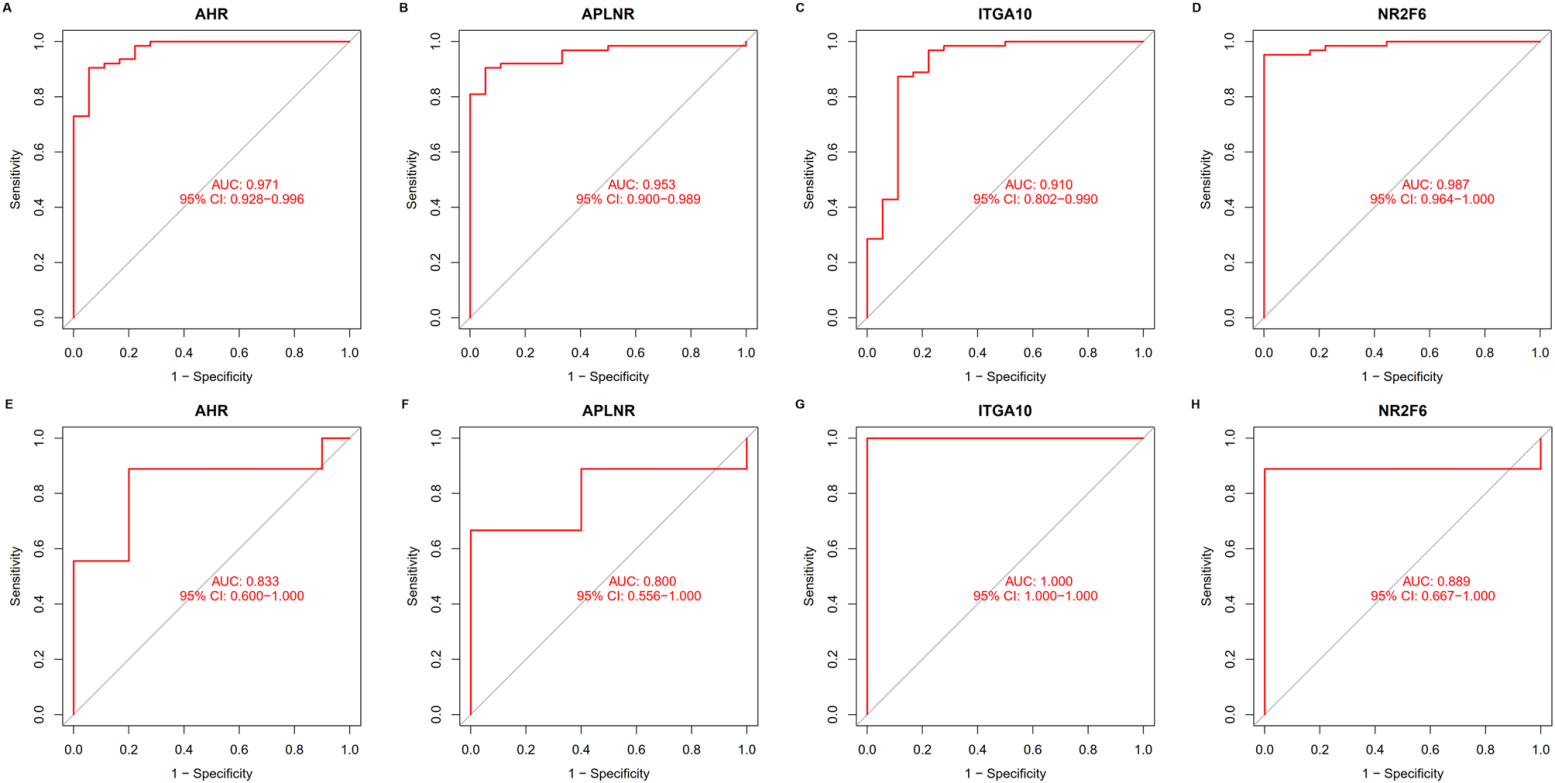
The diagnostic performance of the four feature genes as measured by the ROC curve. (**A**) ROC curve of *AHR* after being fitted to a single variable in the metadata cohort. (**B**) ROC curve of *APLNR* after being fitted to a single variable in the metadata cohort. (**C**) ROC curve of *ITGA10* after being fitted to a single variable in the metadata cohort. (**D**) ROC curve of *NR2F6* after being fitted to a single variable in the metadata dataset. (**E**) ROC curve of *AHR* after being fitted to a single variable in the GSE7084 dataset. (**F**) ROC curve of *APLNR* after being fitted to a single variable in the GSE7084 dataset. (**G**) ROC curve of *ITGA10* after being fitted to a single variable in the GSE7084 dataset. (H) ROC curve of *NR2F6* after being fitted to a single variable in the GSE7084 dataset.

The diagnostic performance of the four feature genes was shown by their capacity to distinguish AAA from the control group, with the area under the ROC curve (AUC) values of 0.971 (95% CI 0.928–0.996) in *AHR* (Fig. 6A), 0.953 (95% CI 0.900–0.989) in *APLNR* (Fig. 6B), 0.910 (95% CI 0.802–0.990) in *ITGA10* (Fig. 6C), and 0.987 (95% CI 0.964–1.000) in *NR2F6* (Fig. 6D). Additionally, the GSE7084 dataset demonstrated a potent capacity for discriminating between the two samples with AUC values of 0.833 (95% CI 0.600–1.000) in *AHR* (Fig. 6E), 0.800 (95% CI 0.556–1.000) in *APLNR* (Fig. 6F), 1.000 (95% CI 1.000–1.000) in *ITGA10* (Fig. 6G), and 0.889 (95% CI 0.667–1.000) in *NR2F6* (Fig. 6H). These suggested that the feature genes had a strong capacity for diagnosis.

### Immune cell infiltration

After downloading the LM22 signature matrix file (see Supplementary File 6), the CIBERSORT bioinformatics method was applied to assess the potential abundance of immune cells utilizing the LM22 file with 1000 permutations. Supplementary File 7 displays the CIBERSORT outcomes.

**Figure 7:**
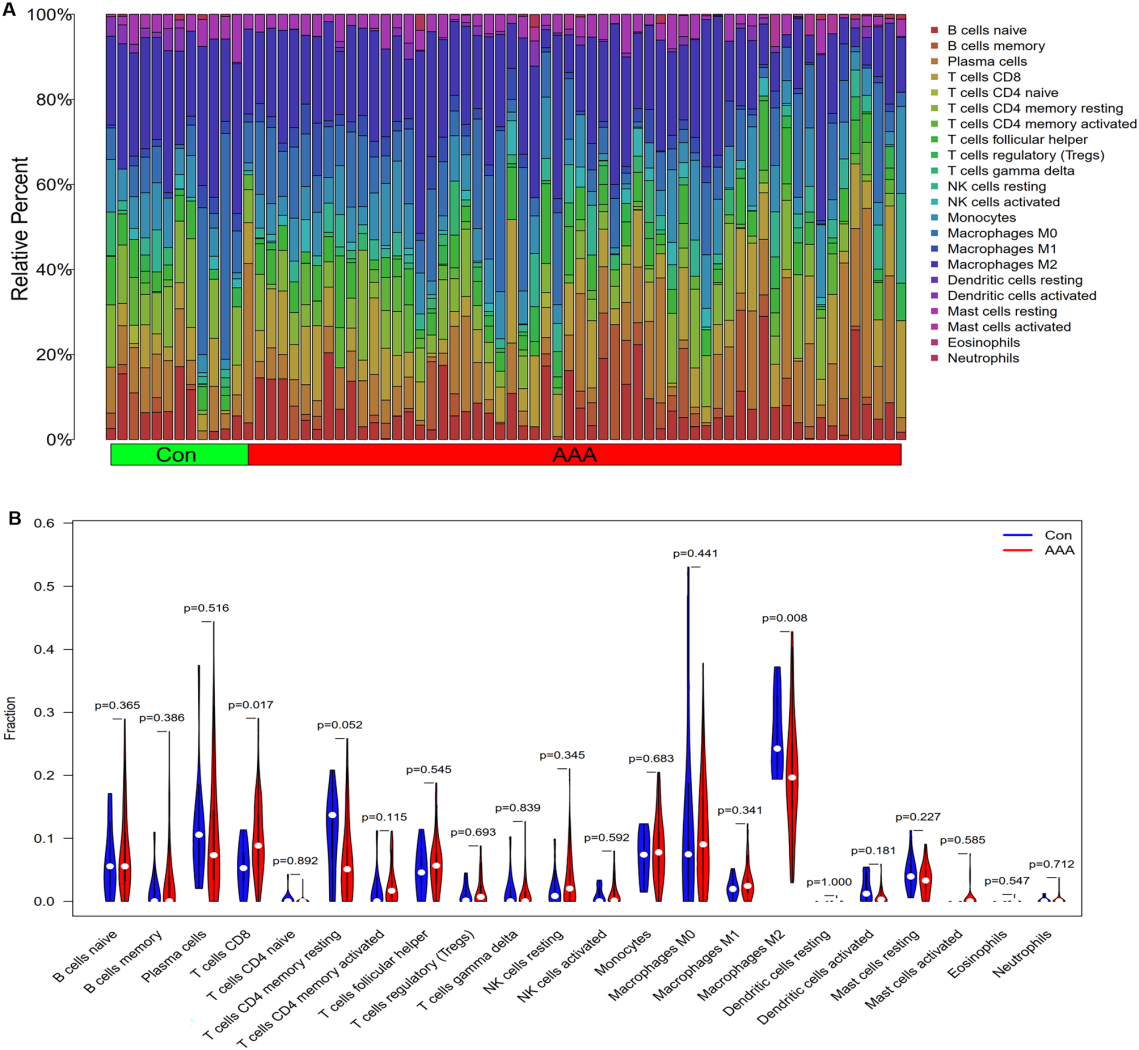
Distribution of infiltrating immune cells. (**A**) Relative abundance of 22 distinct immune cells in AAA samples (AAA) versus control samples (Con). (**B**) Comparison of 22 immune cell subtypes between AAA samples and control samples. Control (Con) and AAA samples (AAA) are represented by blue and red colors, correspondingly.

Figure 7A displays the distribution analysis of the 22 kinds of infiltrating immune cells in the AAA and control groups. Then, we investigated the abundance of immune cells in AAA samples by contrasting them with normal control samples. In comparison to the control group, the proportion of T cells CD8 in AAA was considerably greater (p = 0.017). Conversely, AAA had a substantially lower proportion of Macrophages M2 (p = 0.008) than the normal controls (Fig. 7B). Supplementary Fig. S4 displays the correlation of 22 kinds of infiltrating immune cells.

### Analysis of the association of feature genes with immune cell infiltration

**Figure 8:**
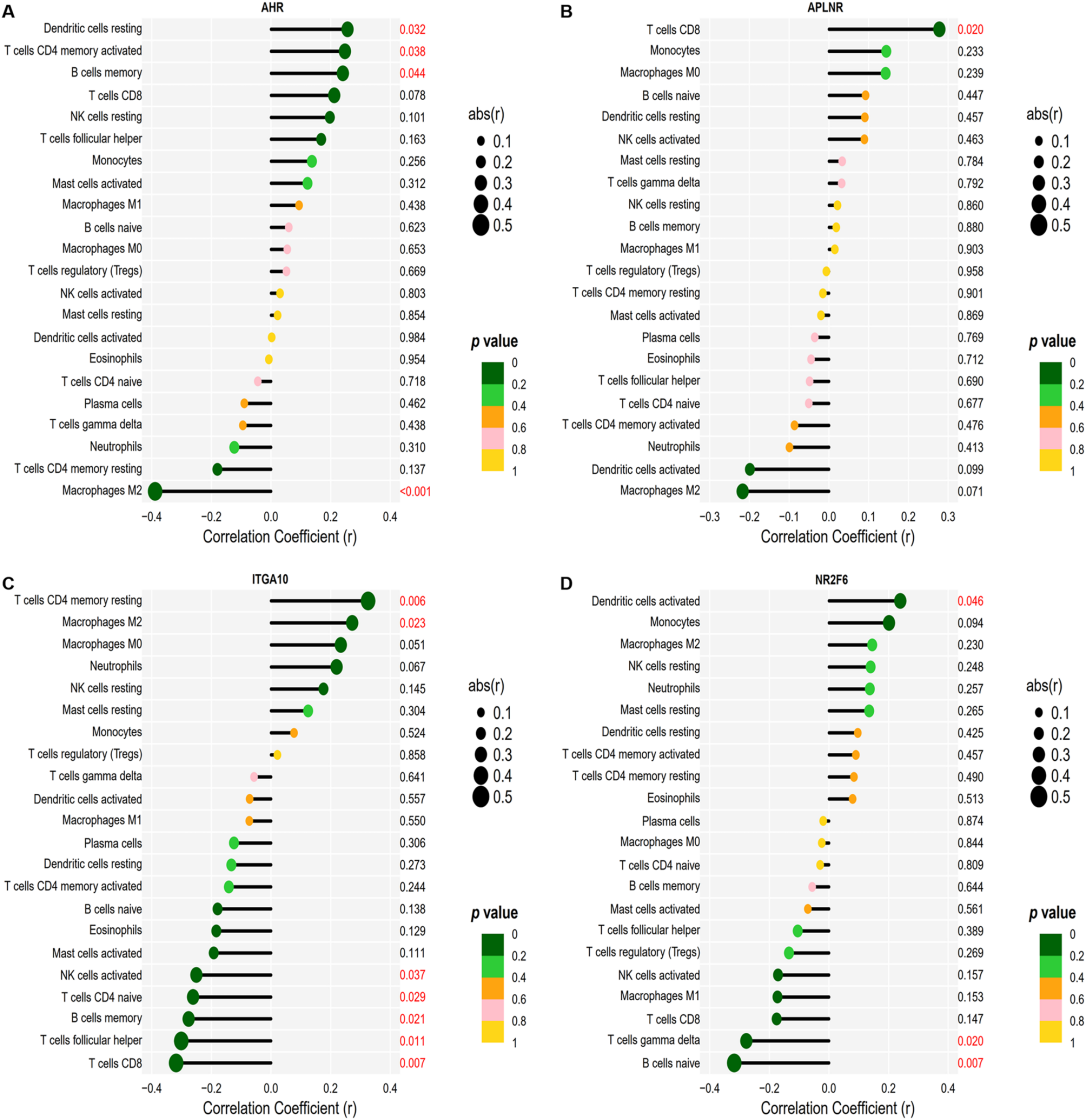
Association between feature genes and immune cell infiltration. (**A**) Association between *AHR* and infiltrating immune cells in AAA. (**B**) Association between *APLNR* and infiltrating immune cells in AAA. (**C**) Association between *ITGA10* and infiltrating immune cells in AAA. (**D**) Association between *NR2F6* and infiltrating immune cells in AAA.

The association between the four feature genes and infiltrating immune cells was examined via Spearman's rank correlation analysis (see Supplementary File 8). *AHR* exhibited a positive association to Dendritic cells resting (r = 0.26, p = 0.032), T cells CD4 memory activated (r = 0.25, p = 0.038), and B cells memory (r = 0.24, p = 0.044) and a negative association with Macrophages M2 (r = − 0.39, p < 0.001) (Fig. 8A). *APLNR* was shown to have a positive association to T cells CD8 (r = 0.28, p = 0.020) (Fig. 8B). *ITGA10* exhibited a positive association with T cells CD4 memory resting (r = 0.33, p = 0.006), Macrophages M2 (r = 0.27, *p* = 0.023) and a negative association with T cells CD8 (r = − 0.32, p = 0.007), T cells follicular helper (r =  − 0.31, p = 0.011), B cells memory (r = − 0.36, p = 0.002), T cells CD4 naive (r = − 0.35, p = 0.002), NK cells activated (r = − 0.28, p = 0.021) (Fig. 8C). *NR2F6* had a positive association to Dendritic cells activated (r = 0.24, p = 0.046) and a negative association to B cells naive (r = − 0.32, p = 0.007), T cells gamma delta (r = − 0.28, p = 0.020) (Fig. 8D).

## Discussion

In this study, microarray datasets of AAA were downloaded from the GEO database. A total of 43 upregulated DEGs and 32 downregulated DEGs was obtained. Function, pathway, disease, and gene set enrichment analyses were performed, in which enrichments were related to inflammation and immune response. *AHR*, APLNR, *ITGA10* and *NR2F6* were defined as feature genes via machine learning algorithms and a validation cohort. The CIBERSORT method was used to quantify the proportions of immune infiltration in samples of AAA and normal tissues. We have predicted *AHR*, *APLNR*, *ITGA10* and *NR2F6* as feature genes of AAA. CD8 + T cells and M2 macrophages correlated with these genes may be involved in the development of AAA.

Patients frequently lose the greatest chance for diagnosis and treatment in the absence of early AAA identification signs, which exacerbates disease progression^3,4^. Therefore, the onset and progression of AAA and the pursuit of treatment options for AAA may benefit from understanding molecular processes of feature genes. According to recent research, immune cells may be crucial to the emergence and progression of AAA^17–19^. Consequently, improving prognosis requires a thorough examination of the link between AAA feature genes and immune cell infiltration.

In recent years, more research has been done on AAA genes and immune infiltration, which has implications for AAA diagnosis and treatment^20–22^. However, few studies have focused on aberrant expression genes linked to immune cell infiltration between AAA and normal tissues. As such, this study explores the feature genes of AAA and evaluates the immune infiltration of these genes into AAA.

First, using microarray technology, we established a metadata cohort with 63 AAA and 18 control samples using two comparable cohorts we obtained from the GEO datasets. In total, 75 DEGs, comprising 32 downregulated and 43 upregulated genes, were identified. Different from the previous studies^23,24^, we found the DEGs were more accurate in the metadata cohort by increasing the sample size. GO functional enrichment illustrated the predominant enrichment of these DEGs in response to toxic substances, regulation of neuroinflammatory response, positive regulation of acute inflammatory response, external side of the plasma membrane, haptoglobin-hemoglobin complex, hemoglobin complex, integrin binding, haptoglobin binding, nuclear receptor activity, implying that these DEGs were strongly linked to inflammation and immunity. The KEGG pathway enrichment confirmed the significant enrichment of DEGs in the IL-17 signaling pathway, malaria, TNF signaling pathway, African trypanosomiasis, rheumatoid arthritis, AGEs-RAGE signaling pathway in diabetic complications, NF-κB signaling pathway, Th17 cell differentiation, transcriptional misregulation in cancer, pertussis. These major pathways were also linked to inflammation and immunity. DO enrichment illustrated that diseases enriched by DEGs were primarily linked to Lyme disease, pulmonary fibrosis, aortic aneurysm, aortic disease, AAA, endometriosis, cervical cancer, interstitial lung disease, cervix carcinoma and agammaglobulinemia and so on. These diseases are associated with AAA, which have some similarity or correlation of pathology to AAA. According to the GSEA data, the enriched pathway was primarily implicated in allograft rejection, ribosomes, Huntington’s disease, dilated cardiomyopathy, type I diabetes mellitus, oxidative phosphorylation, leishmania infection, graft versus host disease, Parkinson’s disease, and autoimmune thyroid disease. These main pathways are also associated with immune responses. These findings generally support earlier findings that the pathophysiology of AAA involves immune response and inflammation^25–28^.

Machine learning algorithms are commonly utilized to discover feature genes and predict disease status given the rapid advancement of science and technology^29,30^. The regularization technique used by the regression analysis method LASSO increases the accuracy of the predictions^31^. SVM is extensively used in disease diagnosis and medical support due to its strong classification and prediction ability. SVM, however, is only particularly good at handling two types of classification issues. Overfitting may be eliminated by using the RFE algorithm. Therefore, investigating the classification accuracy of multiple-oblique situations could be done via the SVM-RFE approach^32^. CIBERSORT is a bioinformatics algorithm that is widely used in the computation of immune infiltration^33,34^. The feature genes among the DEGs of AAA were then identified in this study using the SVM-RFE and LASSO algorithms. Additionally, the function of immune infiltration in AAA was investigated utilizing the CIBERSORT tool.

Five candidate feature genes (*AHR*, *ITGA10*, *PNISR*, *NR2F6*, and *APLNR*) were discovered using SVM-RFE and LASSO algorithms. The GSE7084 dataset was subsequently employed to confirm these five genes' expression levels. In comparison to control tissues, AAA tissues had considerably elevated *AHR* and *APLNR* levels. *ITGA10* and *NR2F6* expression levels in AAA tissues were considerably lowered relative to those in controls. These results were consistent with the differential expression of these genes in the metadata cohort. The variation in the expression of *PNISR* between the two groups was insignificant. Therefore, *AHR*, *APLNR*, *ITGA10*, and *NR2F6* were identified as feature genes to be further investigated. Analysis by ROC and AUC showed that the feature genes were highly capable of diagnosis.

A ligand-activated helix-loop-helix transcription factor known as *AHR*-encoded proteins is implicated in the modulation of physiological reactions to planar aromatic hydrocarbons^35,36^. *APLNR* encodes a member of the gene family for G protein-coupled receptors, which, although linked to the angiotensin receptor, is actually an apelin receptor that inhibits adenylate cyclase activity and reverses the stress effects of angiotensin II (Ang II) by causing a hypertensive response^37,38^. *ITGA10* encodes an integrin alpha chain and is highly expressed in chondrocytes. Integrin is an integral transmembrane glycoprotein consisting of alpha and beta chains that are not covalent bonds. They are involved in cell adhesion and cell-surface-mediated signaling^39,40^. In addition to sequence-specific double-stranded DNA binding, *NR2F6* also promotes transcription factor activity that binds to DNA and is implicated in RNA polymerase II's negative control of transcription^41,42^.

Studies on specific feature genes linked to AAA have been published. *AHR* and its associated signal transduction system are primarily responsible for the inflammatory response, oxidative stress, as well as genetic toxicity of vessel-wall cells^43^. Apelin and its cognate G protein-coupled receptor APLNR constitute a signaling pathway with a positive inotropic effect on cardiac function and a vasodepressor function in the systemic circulation. In a vein graft model, apelin administration reversed Ang II-induced enhancements in neointimal development and vascular remodeling^44^. These findings related feature genes warrant further intensive investigation, while these currently no AAA-related genes worth further exploration.

CIBERSORT was applied to probe the immune infiltrate types in AAA and normal samples. Two immune cell subtypes were consequently shown to be extensively implicated in crucial biological processes of AAA. Comparing AAA tissues to normal tissues, we discovered that the CD8 + T cell infiltration level was elevated while the M2 macrophage infiltration level was lowered in the former, which might have a bearing on the onset and advancement of AAA. Studies about CD8 + T cells and M2 macrophages in AAA have been published in relevant scientific and clinical journals. Experimental AAA was attenuated by *PIAS3* deficiency together with decreased medial elastin disintegration, depletion of smooth muscle cells, accumulation of mural leukocytes, and angiogenesis. *PIAS3*^-/-^ animals had considerably fewer CD8 + T cells in the aortic wall than *PIAS3*^+/+^ mice^45^. Compared with the control group, the CD8 + T cell level was higher in the AAA group^46^. In both in vitro and vivo settings, topiramate administration dramatically facilitated macrophages' conversion from M1 to M2 phenotypes. The M2 macrophage-mediated repair mechanism was strengthened whereas proinflammatory processes were reduced by the M1 macrophages^47^. The onset of rabbit AAA was delayed by IL-10 therapy. The IL-10 treatment's potential molecular mechanism involves facilitating M2 macrophage activation, which suppresses inflammatory processes in aneurysm tissues^48^. The M1/M2 macrophage ratio in AAA tissue is significant, and the predominance of pro-inflammatory cells along with their accompanying markers is seen. By modifying M1/M2 macrophage polarization, elastin-derived peptides enhance AAA onset and progression^49^. By using pathways dependent on macrophage differentiation, pharmacological inhibitors of Notch signaling block the advancement of AAA^50^.

Furthermore, we conducted correlation studies to determine how the four genes were linked to immune cell infiltration. *AHR* linked positively with resting dendritic cells, memory activated CD4 + T cells, memory B cells, and negatively with M2 macrophages. *APLNR* and CD8 + T cells showed a positive association. *ITGA10* was inversely linked to CD8 + T cells, follicular helper T cells, memory B cells, naive CD4 + T cells, and activated NK cells, and positively linked to memory resting CD4 + T cells and M2 macrophages. *NR2F6* had a positive link to activated dendritic cells and an inverse link to naive B cells and gamma delta T cells. According to reports on several diseases, the four genes are linked to these immune cells. M2 macrophages are associated with *AHR*^51,52^. CD8 + T cells are associated with *APLNR*^53^. In-depth experimental studies targeting the link between these genes and these immune cells in AAA deserve further insights, especially the relationship between *AHR* and M2 macrophages, *APLNR* and CD8 + T cells, *ITGA10* and M2 macrophages, *ITGA10* and CD8 + T cells.

While we conducted this study as rigorously as possible, we should acknowledge its limitations. First, the sample size in the metadata cohort still needs to be expanded, even if we combined as many samples as we could from two datasets. Secondly, it is also necessary to increase the validation cohort sample size. Finally, bioinformatics analysis inferred the involvement of four feature genes and immune cell infiltration in AAA, and further experimental studies conducted on larger samples are needed.

In summary, *AHR*, *APLNR*, *ITGA10*, and *NR2F6* were identified as feature genes of AAA. The onset and progression of AAA could be influenced by CD8 + T cells and M2 macrophages that are linked to these genes, which may be used to develop risk predictors and immune interventions.

## Methods

### Microarray data

The National Center for Biotechnology Information (NCBI) GEO database (http://www.ncbi.nlm.nih.gov/geo/) was searched to retrieve the series of matrix files for the GSE57691 and GSE47472 datasets. GEO is an international public repository for high-throughput microarray and next-generation sequence functional genomic data sets submitted by the research community^54^. The GPL10558 Illumina HumanHT-12 V4.0 expression beadchip served as the foundation for both GSE57691 and GSE47472^55,56^. The non-normalized raw data were downloaded to obtain expression matrices using the “lumi” package of R software. The “lumi” package is programmed to perform operations such as Illumina data input, gene annotation, variance stabilization, normalization, and quality control while preprocessing Illumina microarray data^57,58^.

A gene symbol was created for each probe in each dataset using the probe annotation files. The probe with the highest level of expression was determined to represent the gene's final expression value when more than one probe matched to a similar gene symbol. Considering that the two datasets have a common platform and are critical in integrating large sample size data from other datasets, they were merged into a metadata cohort for further study.

Additionally, the validation cohort employed the GSE7084 dataset, which comprised 10 control samples and nine AAA samples and was extracted from the GPL2507 Sentrix Human-6 Expression BeadChip and GPL570 Affymetrix Human Genome U133 Plus 2.0 Array^59–61^.

### Processing data and screening DEGs

Using the combat function of the “SVA” package, the batch effects were preprocessed and eliminated after the two datasets were merged to establish a single metadata cohort^62^. The expression and PCA before and after batch correction was performed to evaluate. Background adjustment, array normalization, and differential expression analysis between AAA and control samples in the metadata dataset or individual datasets were performed utilizing the “limma” package^63^. The thresholds for DEGs were samples exhibiting an adj p value < 0.05 and |log2 FC|> 1. To visualize the expression patterns of the discovered DEGs, a clustered heatmap was created utilizing the “pheatmap” package.

### GO functional and KEGG pathway enrichment analyses of DEGs

To identify the DEGs with the most significant functional and pathway enrichments, we applied the "clusterProfiler" package to conduct GO functional and KEGG pathway enrichment analyses^64,65^. GO functional enrichments encompassed CC, BP and MF. The KEGG database project was initiated in 1995 under the Japanese Human Genome Project, foreseeing the need for a reference resource that would enable understanding of the biological systems, such as the cell and the organism, from genome sequence data^66–68^. The number of enriched genes was denoted by the count value. A q value (adj p value) < 0.05 indicated the significance level.

### DO enrichment analysis and GSEA of DEGs

The “GSEABase” and “DOSE” packages were used to conduct GSEA and DO enrichment analysis for the purpose of identifying disease enrichment in DEGs and the most significant functional terms between AAA and controls^69,70^.

GSEA applied the reference gene set “c2.cp.kegg.v7.0.symbols.gmt” from the Molecular Signatures Database (MSigDB; http://www.gsea-msigdb.org/gsea/msigdb)^71,72^. The ES measures the degree to which a gene set is overrepresented at the top or bottom of a ranked list of genes. Gene set enrichment is indicated by positive ES when it occurs at the top of the list and negative ES when it occurs at its bottom. In GSEA, we set |NES|> 1 and adj p value < 0.25 to indicate the significance of the enrichment.

### Feature gene identification and screening

Two machine learning algorithms were applied to screen AAA-related genes and identify significant characteristic factors. The LASSO improves prediction precision by combining regularization and regression analysis^73^. The LASSO regression algorithm was applied using the “glmnet” package for identifying the genes significantly associated with the discrimination of AAA and normal samples^74^. The supervised machine learning approach referred to as the SVM is frequently employed for classification and regression^75^. The optimum genes from the metadata dataset were chosen using RFE approach to prevent overfitting^76^. Consequently, SVM-RFE was utilized to choose the appropriate features with the aid of the “e1071” and “kernlab” packages in R to determine the gene sets with the highest possible level of discriminative power^77,78^.

Candidates for feature genes were identified as those whose genes overlapped between the two methods. The levels of candidate feature gene expression were then validated in the GSE7084 dataset to identify feature genes.

### Diagnostic significance of identified feature Genes in AAA

The expression data of AAA and control samples in the metadata cohort were used to construct a ROC curve, which was then employed to ascertain the predictive performance of the selected feature genes. The diagnostic potency in differentiating AAA from control samples was evaluated utilizing the AUC value. Subsequently, the AUC value was verified in the GSE7084 dataset.

### Identification of immune cell subtypes

Immune cell infiltration was estimated utilizing the bioinformatics tool CIBERSORT (https://cibersortx.stanford.edu/) to compare the relative proportions of infiltrating immune cell subtypes in AAA and control samples. The Alizadeh Lab and the Newman Lab developed the analytical tool CIBERSORTx, which uses gene expression data for the imputation of gene expression patterns and estimation of the concentration of different cell types within a mixed cell population^33,34^. Based on a reference set of 1000 permutations of the LM22 Signature Matrix file (retrieved from CIBERSORTx), which contains 22 different immune cell subtypes, the potential abundance of immune cells was derived.

Distribution and correlation studies of 22 different kinds of invading immune cells were carried out utilizing the “corrplot” package in R. The “corrplot” package is a graphical display of a correlation matrix, confidence interval. Furthermore, to illustrate the variations in immune cell infiltration levels between the AAA and control samples, violin plots were created utilizing the “vioplot” package.

### Analysis of the association between feature genes and immune cell infiltration

Spearman's rank correlation test was conducted to examine the association between the discovered feature genes and the abundance of infiltrating immune cells. The resultant relationships were displayed using a charting approach with the “ggplot2” package. The “ggplot2” package is a commonly used data visualization tool to create a variety of high-quality graphs.

### Statistical analysis

R (version 4.0.3) software and associated packages were applied for all analyses of statistical data. Continuous variables between groups were compared utilizing either the Mann–Whitney U test for data conforming to an abnormal distribution or the Student's t test for normally distributed data. The effect size for Mann–Whitney U or Student's t tests was also calculate via Social Science Statistics (https://www.socscistatistics.com/effectsize/default3.aspx). The “glmnet” package was adopted to conduct the LASSO regression analysis, while the “e1071” and “kernlab” packages were adopted to conduct the SVM-RFE algorithm. Additionally, the diagnostic significance of the identified feature genes was ascertained utilizing the ROC curve analysis and AUC value. Spearman's correlation was conducted to elucidate the association between the expression of feature genes and immune cell infiltration. The absolute value of the correlation coefficient above 0.7 was considered as a strong correlation and the absolute value of the correlation coefficient between 0.3 and 0.7 was considered as a moderate correlation. All statistical tests were two-sided with p < 0.05 serving as the significance threshold. When screening DEGs between AAA and control samples, the thresholds were an adj p value < 0.05 and |log2FC|> 1. A q value < 0.05 was recognized as statistically significant in GO functional enrichment, KEGG pathway, and DO enrichment analyses. In GSEA, the significance enrichment criteria were |NSE|> 1 and adj p < 0.25.

### Supplementary Information

Supplementary Information.

## Data Availability

The datasets and R codes generated and/or analyzed during the current study are available from the corresponding author upon reasonable request. Main data generated or analyzed during this study are included in this published article (and its Supplementary Information files).
