# Supplementary material for: Predicting feature genes correlated with immune infiltration in patients with abdominal aortic aneurysm based on machine learning algorithms
Source: Sci Rep. 2024 Mar 2;14:5157. doi: 10.1038/s41598-024-55941-6 (PMC10908806; doi:10.1038/s41598-024-55941-6)
Supplement: Supplementary file 1 — Supplementary Information. [file 41598_2024_55941_MOESM1_ESM.zip › Supplementary Figure.pdf]

Figure S1

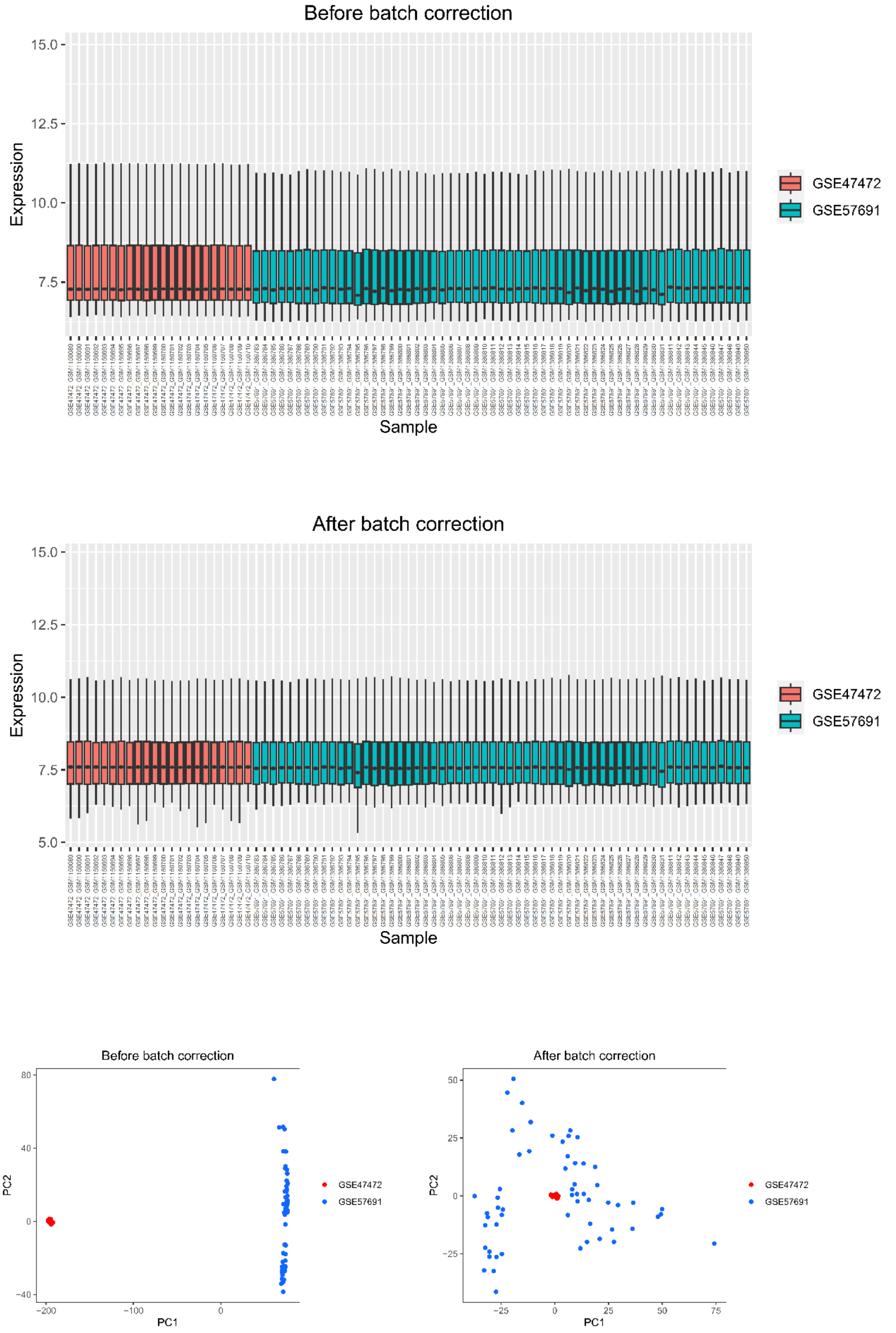

The expression and principal component analysis before and after batch correction showed that the baseline of the two datasets were fairly consistent and further normalized.

Figure S2

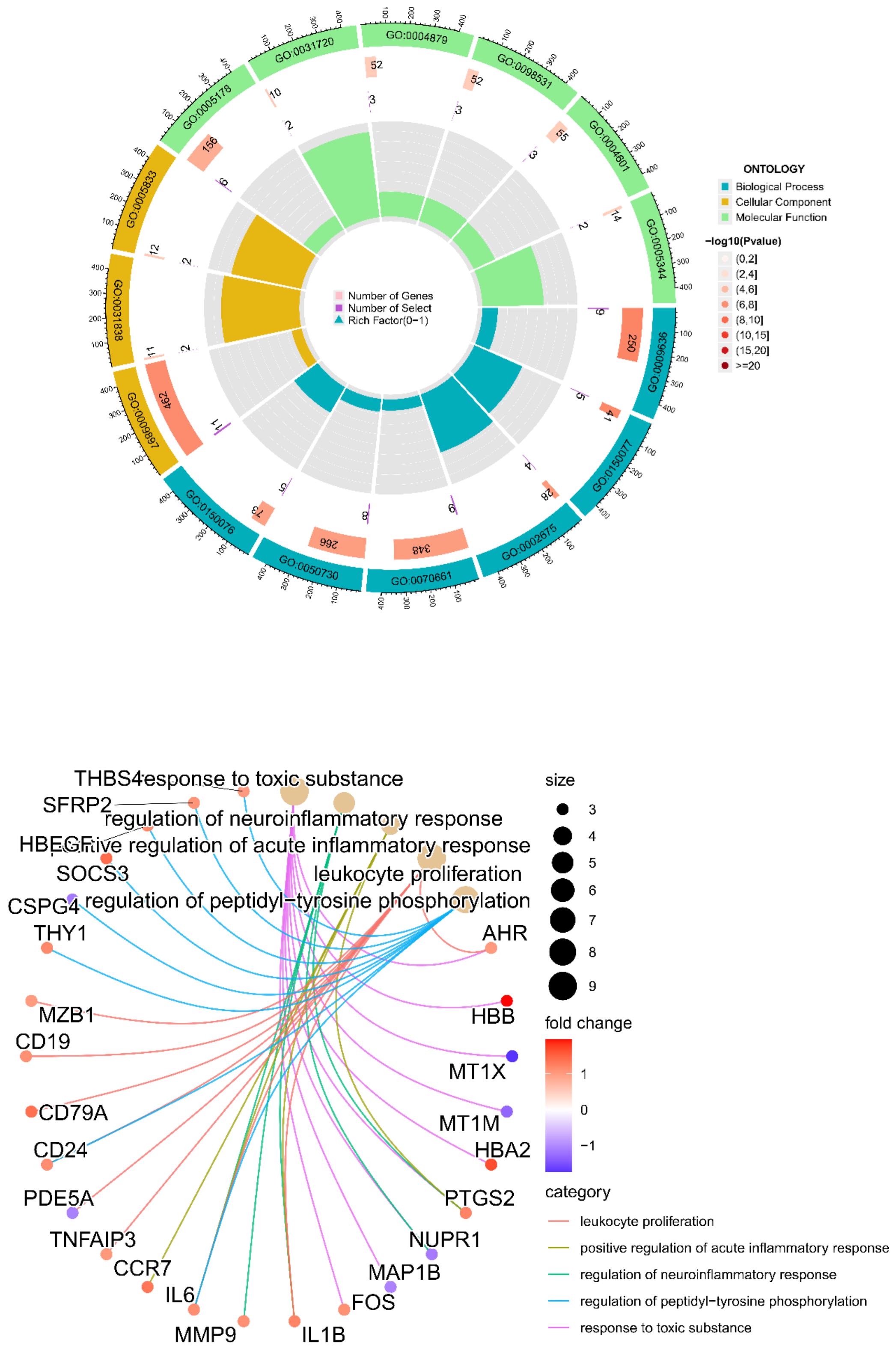

The GO plots represented as a network to understand the connection between most prominent GO terms

Figure S3

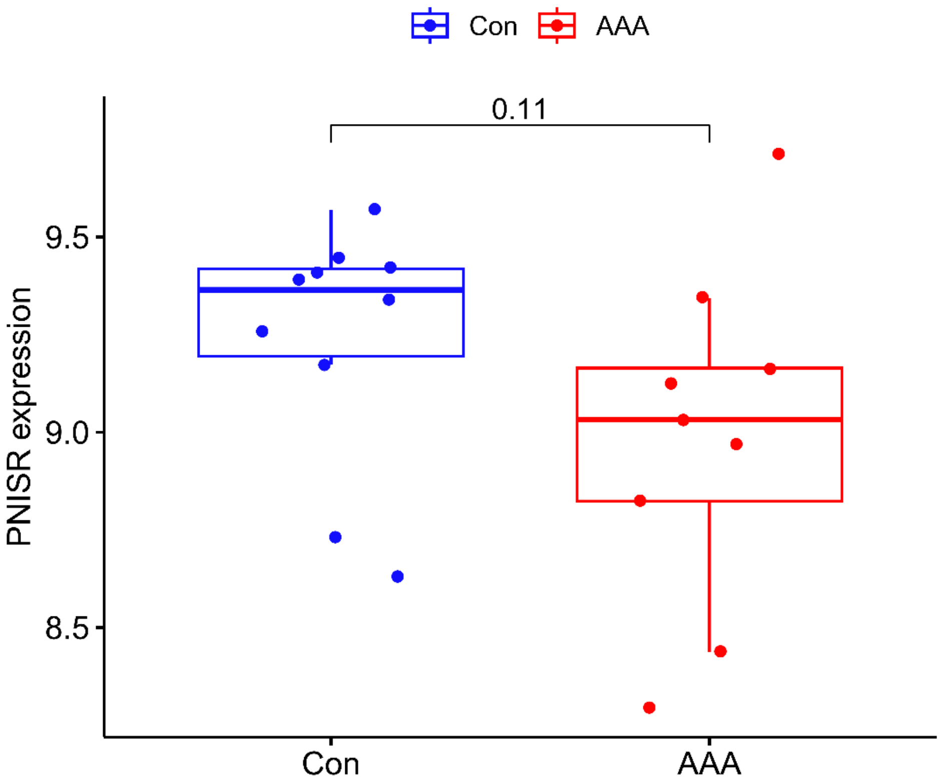

Comparison of the gene expression levels in AAA (AAA) samples to those in control samples (Con). PNISR expression levels did not differ significantly across AAA and control samples.

Figure S4

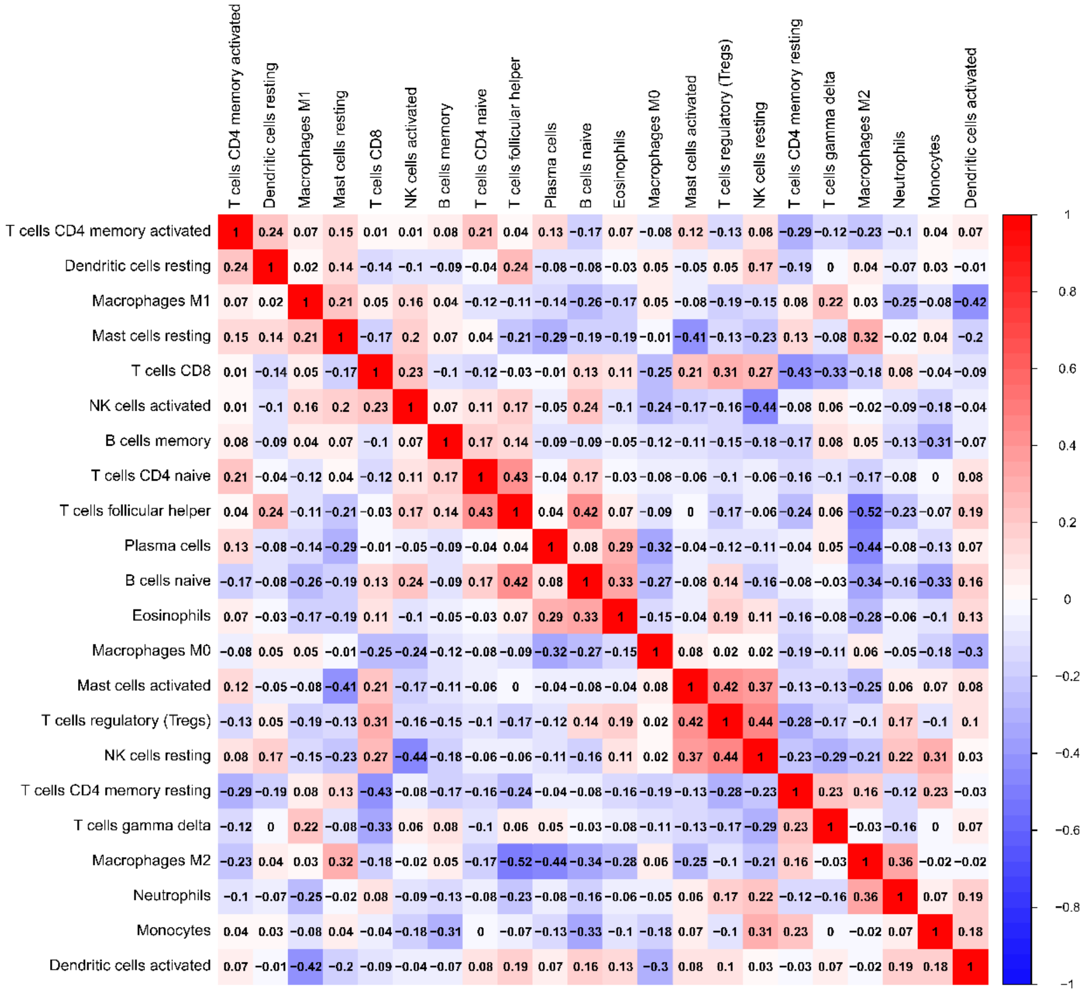

The correlation of 22 kinds of infiltrating immune cells.
