# Supplementary material for: Predicting feature genes correlated with immune infiltration in patients with abdominal aortic aneurysm based on machine learning algorithms
Source: Sci Rep. 2024 Mar 2;14:5157. doi: 10.1038/s41598-024-55941-6 (PMC10908806; doi:10.1038/s41598-024-55941-6)
Supplement: Supplementary file 1 — Supplementary Information. [file 41598_2024_55941_MOESM1_ESM.zip › Supplementary File 1A The clinical overall design of the three datasets.pdf]

Series GSE47472	Query DataSets for GSE47472
Status	Public on May 30, 2013
Title	Differential gene expression in the proximal neck of human abdominal aortic aneurysm
Organism	Homo sapiens
Experiment type	Expression profiling by array
Summary	The aim of this study was to assess the gene expression profile of biopsies obtained from the neck of human AAAs.
Overall design	Genome-wide expression analysis of AAA neck specimen obtained from 14 patients with AAA (mean maximum aortic diameter=62.6±18.0 mm). Relative aortic gene expression was compared with that of 8 control aortic specimen of organ donors.
Contributor(s)	Gäbel G, Lindeman JH, Walker PJ, Golledge J, Biros E
Citation(s)	• Biros E, Moran CS, Rush CM, Gäbel G et al. Differential gene expression in the proximal neck of human abdominal aortic aneurysm. *Atherosclerosis* 2014 Mar;233(1):211-8. PMID: 24529146
Submission date	May 29, 2013
Last update date	Dec 15, 2021
Contact name	Erik Biros
Organization name	James Cook University
Street address	James Cook Drive
City	Townsville
ZIP/Postal code	4811
Country	Australia
Platforms (1)	GPL10558 Illumina HumanHT-12 V4.0 expression beadchip
Samples (22)	GSM1150689 AAA neck (Sample 1)
	GSM1150690 AAA neck (Sample 2)
	GSM1150691 AAA neck (Sample 3)
	GSM1150692 AAA neck (Sample 4)
	GSM1150693 AAA neck (Sample 5)
	GSM1150694 AAA neck (Sample 6)
	GSM1150695 AAA neck (Sample 7)
	GSM1150696 AAA neck (Sample 8)
	GSM1150697 AAA neck (Sample 9)
	GSM1150698 AAA neck (Sample 10)
	GSM1150699 AAA neck (Sample 11)

GSM1150700 AAA neck (Sample 12)

GSM1150701 AAA neck (Sample 13)

GSM1150702 AAA neck (Sample 14)

GSM1150703 Donor (Sample 15)

GSM1150704 Donor (Sample 16)

GSM1150705 Donor (Sample 17)

GSM1150706 Donor (Sample 18)

GSM1150707 Donor (Sample 19)

GSM1150708 Donor (Sample 20)

GSM1150709 Donor (Sample 21)

GSM1150710 Donor (Sample 22)

Relations

BioProject PRJNA205833

Analyze with GEO2R

Download family	Format
SOFT formatted family file(s)	SOFT
MINiML formatted family file(s)	MINiML
Series Matrix File(s)	TXT

Supplementary file	Size	Download	File type/resource
GSE47472_RAW.tar	26.2 Mb	(http)(custom)	TAR
GSE47472_Rawdata_GEO_AAA_Neck.txt.gz	3.7 Mb	(ftp)(http)	TXT

Processed data included within Sample table

Series GSE57691	Query DataSets for GSE57691
Status	Public on Jun 30, 2015
Title	Differential gene expression in human abdominal aortic aneurysm and atherosclerosis
Organism	Homo sapiens
Experiment type	Expression profiling by array
Summary	The aim of this study was to assess the relative gene expression in human AAA and AOD.
Overall design	Genome-wide expression analysis of abdominal aortic aneurysm (AAA) and aortic occlusive disease (AOD) specimens obtained from 20 patients with small AAA (mean maximum aortic diameter=54.3±2.3 mm), 29 patients with large AAA (mean maximum aortic diameter=68.4±14.3 mm), and 9 AOD patients (mean maximum aortic diameter=19.6±2.6 mm). Relative aortic gene expression was compared with that of 10 control aortic specimen of organ donors.
Contributor(s)	Gäbel G, Lindeman JH, Walker PJ, Golledge J, Biros E
Citation(s)	• Biros E, Gäbel G, Moran CS, Schreurs C et al. Differential gene expression in human abdominal aortic aneurysm and aortic occlusive disease. *Oncotarget* 2015 May 30;6(15):12984-96. PMID: 25944698
Submission date	May 15, 2014
Last update date	Sep 06, 2018
Contact name	Erik Biros
Organization name	James Cook University
Street address	James Cook Drive
City	Townsville
ZIP/Postal code	4811
Country	Australia
Platforms (1)	GPL10558 Illumina HumanHT-12 V4.0 expression beadchip
Samples (68)	GSM1386783 small AAA_Sample 1
	GSM1386784 small AAA_Sample 2
	GSM1386785 small AAA_Sample 3
	GSM1386786 small AAA_Sample 4
	GSM1386787 small AAA_Sample 5
	GSM1386788 small AAA_Sample 6
	GSM1386789 small AAA_Sample 7
	GSM1386790 small AAA_Sample 8
	GSM1386791 small AAA_Sample 9

GSM1386792 small AAA_Sample 10

GSM1386793 small AAA_Sample 11

GSM1386794 small AAA_Sample 12

GSM1386795 small AAA_Sample 13

GSM1386796 small AAA_Sample 14

GSM1386797 small AAA_Sample 15

GSM1386798 small AAA_Sample 16

GSM1386799 small AAA_Sample 17

GSM1386800 small AAA_Sample 18

GSM1386801 small AAA_Sample 19

GSM1386802 small AAA_Sample 20

GSM1386803 large AAA_Sample 21

GSM1386804 large AAA_Sample 22

GSM1386805 large AAA_Sample 23

GSM1386806 large AAA_Sample 24

GSM1386807 large AAA_Sample 25

GSM1386808 large AAA_Sample 26

GSM1386809 large AAA_Sample 27

GSM1386810 large AAA_Sample 28

GSM1386811 large AAA_Sample 29

GSM1386812 large AAA_Sample 30

GSM1386813 large AAA_Sample 31

GSM1386814 large AAA_Sample 32

GSM1386815 large AAA_Sample 33

GSM1386816 large AAA_Sample 34

GSM1386817 large AAA_Sample 35

GSM1386818 large AAA_Sample 36

GSM1386819 large AAA_Sample 37

GSM1386820 large AAA_Sample 38

GSM1386821 large AAA_Sample 39

GSM1386822 large AAA_Sample 40

GSM1386823 large AAA_Sample 41

GSM1386824 large AAA_Sample 42

GSM1386825 large AAA_Sample 43

GSM1386826 large AAA_Sample 44

GSM1386827 large AAA_Sample 45

GSM1386828 large AAA_Sample 46

GSM1386829 large AAA_Sample 47

GSM1386830 large AAA_Sample 48

GSM1386831 large AAA_Sample 49

GSM1386832 AOD_Sample 50

GSM1386833 AOD_Sample 51

GSM1386834 AOD_Sample 52

GSM1386835 AOD_Sample 53

GSM1386836 AOD_Sample 54

GSM1386837 AOD_Sample 55

GSM1386838 AOD_Sample 56

GSM1386839 AOD_Sample 57

GSM1386840 AOD_Sample 58

GSM1386841 Donor_Sample 59

GSM1386842 Donor_Sample 60

GSM1386843 Donor_Sample 61

GSM1386844 Donor_Sample 62

GSM1386845 Donor_Sample 63

GSM1386846 Donor_Sample 64

GSM1386847 Donor_Sample 65

GSM1386848 Donor_Sample 66

GSM1386849 Donor_Sample 67

GSM1386850 Donor_Sample 68

Relations

BioProject PRJNA247729

Analyze with GEO2R

**Download family**	**Format**
SOFT formatted family file(s)	SOFT
MINiML formatted family file(s)	MINiML
Series Matrix File(s)	TXT

Supplementary file	Size	Download	File type/resource
GSE57691_RAW.tar	26.2 Mb	(http)(custom)	TAR
GSE57691_non-normalized_data.txt.gz	10.6 Mb	(ftp)(http)	TXT

Processed data included within Sample table

Series GSE7084	Query DataSets for GSE7084
Status	Public on Jul 26, 2007
Title	Expression data from the abdominal aorta and abdominal aortic aneurysm
Organism	Homo sapiens
Experiment type	Expression profiling by array
Summary	Global gene expression information that can be used to identify pathways involved in the pathophysiology of disease.<br>We used microarrays to identify which genes are expressed in either the abdominal aorta (control) or in abdominal aortic aneurysms (case), and also which genes may be differential between the two tissue states.<br>Keywords: Characterization of expression in both diseased and non-diseased abdominal aortas.
Overall design	Abdominal aortas and abdominal aortic aneurysms were obtained from either autopsy within 24 hours of death (control tissue) or surgical prooceedures (aneurysms). RNA was isolated and run on two platforms either as pools or as the individual samples contributing to the pools.
Contributor(s)	Tromp G, Kuivaniemi H, Lenk GM, Weinsheimer S
Citation(s)	• Lenk GM, Tromp G, Weinsheimer S, Gatalica Z et al. Whole genome expression profiling reveals a significant role for immune function in human abdominal aortic aneurysms. *BMC Genomics* 2007 Jul 16;8:237. PMID: 17634102<br>• Hinterseher I, Erdman R, Donoso LA, Vrabec TR et al. Role of complement cascade in abdominal aortic aneurysms. *Arterioscler Thromb Vasc Biol* 2011 Jul;31(7):1653-60. PMID: 21493888<br>• Pahl MC, Erdman R, Kuivaniemi H, Lillvis JH et al. Transcriptional (ChIP-Chip) Analysis of ELF1, ETS2, RUNX1 and STAT5 in Human Abdominal Aortic Aneurysm. *Int J Mol Sci* 2015 May 18;16(5):11229-58. PMID: 25993293
Submission date	Feb 20, 2007
Last update date	Jun 10, 2019
Contact name	Gerard Tromp
E-mail(s)	gerard.tromp@sanger.med.wayne.edu
Phone	313-577-8773
Fax	313-577-5218
Organization name	Wayne State University School of Medicine
Department	Center for Molecular Medicine and Genetics
Street address	3309 Scott Hall, 540 East Canfield Ave
City	Detroit
State/province	MI
ZIP/Postal code	48201

Country	USA

Platforms (2)	GPL570 [HG-U133_Plus_2] Affymetrix Human Genome U133 Plus 2.0 Array<br>GPL2507 Sentrix Human-6 Expression BeadChip
Samples (19)	GSM170550 Abdominal Aortic Aneurysm1-F<br>GSM170551 Abdominal Aortic Aneurysm1-M<br>GSM170552 Abdominal Aortic Aneurysm2-F<br>GSM170553 Abdominal Aortic Aneurysm2-M<br>GSM170554 Abdominal Aortic Aneurysm3-M<br>GSM170555 Abdominal Aortic Aneurysm4-M<br>GSM170556 Control Abdominal Aorta 1-F<br>GSM170557 Control Abdominal Aorta 1-M<br>GSM170558 Control Abdominal Aorta 2-F<br>GSM170559 Control Abdominal Aorta 2-M<br>GSM170560 Control Abdominal Aorta 3-F<br>GSM170561 Control Abdominal Aorta 3-M<br>GSM170562 Control Abdominal Aorta 4-M<br>GSM170563 Abdominal Aortic Aneurysm Male Pool I<br>GSM170564 Control Abdominal Aorta Male Pool I<br>GSM170565 Abdominal Aortic Aneurysm Male Pool A<br>GSM170566 Control Abdominal Aorta Male Pool A<br>GSM170567 Abdominal Aortic Aneurysm Female Pool<br>GSM170568 Control Abdominal Aorta Female Pool

Relations

BioProject	PRJNA98459

Analyze with GEO2R

Download family	Format
SOFT formatted family file(s)	SOFT
MINiML formatted family file(s)	MINiML
Series Matrix File(s)	TXT

Supplementary file	Size	Download	File type/resource
GSE7084_RAW.tar	8.2 Mb	(http)(custom)	TAR (of TXT)

Processed data included within Sample table
